# Retrospective Review of the Efficacy of Droperidol Compared to Prochlorperazine for Headache Management in the Emergency Department

**DOI:** 10.7759/cureus.39848

**Published:** 2023-06-01

**Authors:** Gabrielle K Driller, Adrianne Remigio, Jason Teng, Andrea Fang, Jonathan Hootman, Allen Chang

**Affiliations:** 1 Pharmacy, Stanford Health Care, Stanford, USA; 2 Emergency Medicine, Virginia Hospital Center, Arlington, USA; 3 Emergency Medicine, Stanford University School of Medicine, Stanford, USA; 4 Critical Care Medicine, Stanford University School of Medicine, Stanford, USA; 5 Emergency Medicine, Kaiser North Valley, Roseville, USA

**Keywords:** headache, ketorolac tromethamine, prochlorperazine, droperidol, migraine

## Abstract

Introduction

Headaches are a common presentation to the emergency department, representing approximately 3% of visits. The standard treatment of headaches has consisted of either monotherapy with an antidopaminergic agent or combination therapy with an antidopaminergic agent, a non-steroidal anti-inflammatory drug (NSAID), and diphenhydramine. Although droperidol is an antidopaminergic medication, it previously was not widely used in the treatment of headaches due to safety concerns. Given its pharmacokinetics, droperidol may provide faster relief in migrainous headaches compared to more commonly used antidopaminergic agents.

Methods

We conducted a single-center retrospective chart review to examine the impact of droperidol compared to other standard migraine therapies on pain scores. The study consisted of three treatment arms: droperidol monotherapy, a droperidol bundle (droperidol and ketorolac), and a prochlorperazine bundle (prochlorperazine and ketorolac). Patients who received medications in treatment arms and who had an encounter diagnosis including either “headache” or “migraine” were included. Patients were excluded if under 18 years of age, imprisoned, pregnant, or received potentially migraine-altering medications prior to the first documented pain score. The primary outcome was a mean reduction in pain scores. Secondary outcomes included length of emergency department stay, rates of inpatient admission, need for rescue therapies, and adverse events.

Results

A total of 361 droperidol orders were reviewed, of which 79 met the inclusion criteria. Of those included, 30 orders were within the droperidol monotherapy arm, 19 were within the droperidol bundle arm, and 30 were within the prochlorperazine bundle arm. There were no significant differences in reduction of pain scores, emergency department length of stay, rates of inpatient admission, rates of rescue therapy, or adverse events between the three treatment arms.

Conclusion

In this study, we found no statistical difference in migraine treatment efficacy between droperidol monotherapy and droperidol and prochlorperazine-based bundle therapies. Further studies are needed with larger sample sizes and predefined timing between pain score charting and medication administration.

## Introduction

Headaches are a common presentation to the emergency department and account for more than 3.5 million visits a year, representing approximately 3% of all emergency visits [1,2]. Effective treatment of headaches is critical to the care and disposition of these patients. The standard treatment of headaches in the emergency department (ED) usually consists of monotherapy with an antidopaminergic agent or combination therapy with an antidopaminergic agent, a non-steroidal anti-inflammatory drug (NSAID), and diphenhydramine [3]. Antidopaminergic agents chosen for the treatment of migraines include prochlorperazine, metoclopramide, and chlorpromazine [4,5]. Other treatment options for migraines include acetaminophen, triptans, ergotamine preparations, caffeine, magnesium, corticosteroids, valproic acid, nerve blocks, ketamine, propofol, lasmiditan, and cGRP antagonists [4-6]. Within the Expert Clinical Management series of the Annals of Emergency Medicine, the suggested treatment of refractory acute migrainous headaches consists of an antidopaminergic agent with diphenhydramine [3]. In case the symptoms continue, the suggested treatment algorithm recommends a second dose of the antidopaminergic accompanied by ketorolac. For migraines that continue to be resistant to treatment, providers can then consider opioids, occipital nerve blocks, and/or dihydroergotamine [3]. A fast, effective cocktail therapy would be ideal to improve the length of stay and ED throughput. Droperidol is an antidopaminergic medication that has regained popularity in recent years after numerous studies demonstrated that initial concerns regarding cardiac complications were largely unfounded at typical doses [7,8]. Due to its pharmacokinetic profile, droperidol may provide faster relief than other antidopaminergic medications with a comparatively slower onset in treating migrainous headaches.

When compared to placebo, droperidol has been shown to be more effective at reducing pain due to migraine at doses ranging from 2.75 milligrams (mg) to 8.25 mg [9,10]. Droperidol at doses of 2.5 mg to 5 mg given either intravenously (IV) or intramuscularly (IM) has been demonstrated to be at least as effective as alternative antidopaminergic agents such as prochlorperazine and olanzapine [10]. The results from one randomized controlled trial suggest that droperidol may even be more effective than prochlorperazine in treating headache-associated pain [11].

The most common adverse effects reported with the use of droperidol for the treatment of migraines are drowsiness and extrapyramidal symptoms [10,12]. The incidence of akathisia due to droperidol has been suggested to be similar to that seen with prochlorperazine [13]. In the randomized studies reviewed, droperidol at doses under 10 mg was not linked to any cases of QTc prolongation [9,14]. However, dose-dependent QTc prolongation may be associated with droperidol at doses at and above 10 mg [14].

Current evidence has not compared droperidol to the more common combination of medications, such as prochlorperazine with an NSAID, typically ordered to treat migraines. The purpose of this study is to compare the efficacy of droperidol to the standard migraine “bundle” in reducing pain due to headaches. We also aim to compare the safety, time to discharge, and need for rescue therapy between each of our three treatment groups. This article was previously presented as a meeting abstract at the 2022 Western States Conference on May 17, 2022.

## Materials and methods

We conducted a single-center retrospective chart review of 361 medication orders associated with emergency department encounters spanning from January 2017 to July 2021 to examine the impact of droperidol as compared to other standard migraine therapies on pain scores. The study consisted of three treatment arms: droperidol monotherapy; a droperidol bundle, including droperidol and ketorolac; and a prochlorperazine bundle, consisting of prochlorperazine and ketorolac (Table 1).

**Table 1:** Treatment arms - Concomitant medications that may be included: acetaminophen, diphenhydramine, magnesium, lidocaine, ketamine, lorazepam, baclofen, gabapentin, pregabalin, aspirin, ondansetron, dexamethasone - Concomitant medications that require case exclusion: NSAIDs (except aspirin and ketorolac), metoclopramide, opioids, triptans, dihydroergotamine, butalbital/acetaminophen/caffeine NSAIDs: non-steroidal anti-inflammatory drugs

Droperidol bundle	Prochlorperazine bundle	Droperidol monotherapy
Droperidol	Prochlorperazine	Droperidol
Ketorolac	Ketorolac	

Charts were included if the patient received the study medications within one of the three treatment groups, had an emergency department encounter diagnosis including either “headache” or “migraine,” and did not meet exclusion criteria. Exclusion criteria consisted of age under 18 years, imprisonment at the time of treatment, pregnancy, documentation of self-treatment with dihydroergotamine or triptan medications prior to emergency department arrival, prior contraindications or hypersensitivities to any of the study medications, suspicion of malignant headache, or documentation of the administration of one of the following medications within two hours of the first documented pain score: NSAIDs (except aspirin or ketorolac), metoclopramide, opioids, dihydroergotamine, butalbital/acetaminophen/caffeine, or triptans.

The primary outcome was a mean reduction in pain scores. Secondary outcomes consisted of the length of emergency department stay, rates of inpatient admission, need for rescue therapies (as defined by the administration of anti-migraine medications following the administration of study medications), and adverse events (including extrapyramidal symptoms, dystonic reactions, akathisia, and QTc prolongation).

One-way analysis of variance (ANOVA) testing was conducted to assess for significant endpoint differences between the three treatment arms. When comparing the two treatment arms, two-sample paired T-tests were conducted. A p-value of less than 0.05 was considered to be statistically significant. This study was approved by the institutional review board at Stanford University on November 5, 2021 (protocol #62846).

## Results

A total of 361 droperidol orders were reviewed, of which 79 met the inclusion criteria. Within the 79 included orders, 30 orders were within the droperidol monotherapy arm, 19 were within the droperidol bundle arm, and 30 were within the prochlorperazine bundle arm.

There were no statistically significant differences in gender distribution or weight between the three treatment arms at baseline. However, there was a statistically significant difference in age between the three treatment arms, with the median age in the droperidol monotherapy arm being nine years older than that in the droperidol bundle and prochlorperazine bundle arms (Table 2). The mean doses of each treatment agent are shown in Table 2. All doses of prochlorperazine and ketorolac were given intravenously. All doses of droperidol were given intravenously, except for one dose, which was given intramuscularly.

**Table 2:** Baseline characteristics of patients in the three treatment arms

		Droperidol monotherapy	Droperidol bundle	Prochlorperazine bundle	P-value
Sex (n, %)	Male	11 (37%)	3 (16%)	8 (27%)	0.28
	Female	19 (63%)	16 (84%)	22 (73%)	
Age (median, years)		45.42	36.64	36.15	0.01
Weight (median, kg)		78.6	77.1	80.95	0.91
Droperidol dose (mean, mg)		2.32	2.25	---	---
Ketorolac dose (mean, mg)		---	15.8	17.3	---
Prochlorperazine dose (mean, mg)		---	---	6.25	---

Patients in the droperidol monotherapy and droperidol bundle arms had mean pain score reductions of 3.6 and 5.0, respectively. Patients in the prochlorperazine bundle had a mean pain score reduction of 5.3. The difference in pain score reduction or time between pain score collection and study medication administration was not found to be statistically significant between the three treatment arms (Table 3).

**Table 3:** Pain score reduction and time between pain scores and medication administration

	Droperidol monotherapy	Droperidol bundle	Prochlorperazine bundle	P-value
Pain score reduction (mean)	3.57	4.95	5.33	0.07
Time between 1st pain score and study medication administration (median, minutes)	32.5	68	41	0.11
Time between 2nd pain score and study medication administration (median, minutes)	59	86	67.5	0.07

**Figure 1:**
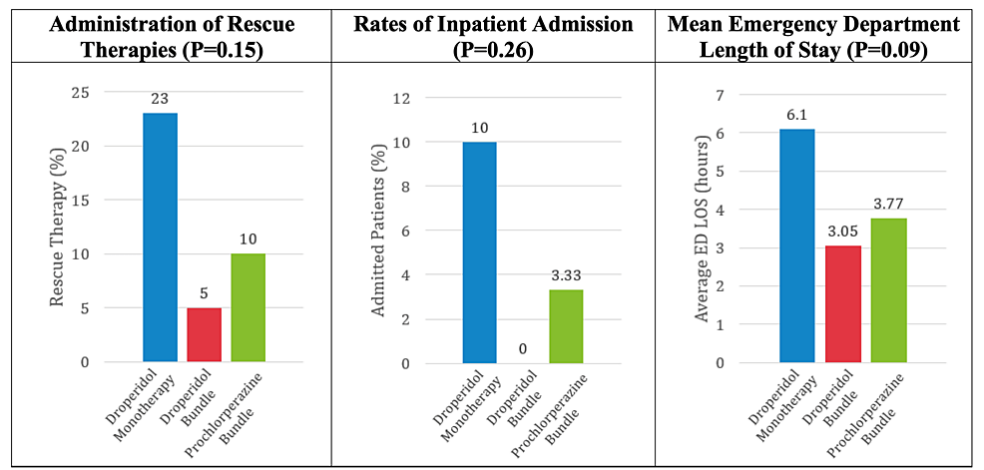
Comparison of need for rescue therapy, inpatient admission rates, and ED length of stay between the treatment arms

When studying the secondary outcomes, there was no statistically significant difference regarding the administration of rescue therapies, rates of inpatient admission, or emergency department length of stay between the three treatment arms (Figure 1). However, there was a trend toward greater rates of rescue therapy, rates of inpatient admission, and emergency department length of stay in the droperidol monotherapy group.

No patients in the droperidol monotherapy and prochlorperazine bundle arms suffered adverse reactions. Five percent of patients in the droperidol bundle did; however, this difference was not found to be statistically significant. There was not a statistically significant difference in QTc prolongation between the three treatment arms (Table 4).

**Table 4:** Safety endpoints - Adverse reactions include extrapyramidal symptoms, dystonic reactions, and akathisia

	Droperidol monotherapy	Droperidol bundle	Prochlorperazine bundle	P-value
Adverse reactions (%)*	0	5	0	0.21
QTc prolongation (>500 msec, %)	3.33	0	0	0.99

## Discussion

Droperidol was not widely referenced as an anti-migraine therapy prior to 2019 due to safety concerns, unlike prochlorperazine, which was commonly included in migraine therapy reviews [4,6]. However, after the risk of cardiac effects with standard doses of droperidol was found to be overestimated, droperidol has reemerged as a potential anti-migraine medication [5,7,8]. Further, droperidol’s place in the treatment of migraines is recognized by the Annals of Emergency Medicine’s clinical management review where antidopaminergics, including droperidol, metoclopramide, and prochlorperazine, are all stated to have anti-migraine activity [3]. Based on our study’s results, we were unable to detect a significant difference in migraine-related pain reduction between the use of prochlorperazine and droperidol. While the results demonstrated a 0.38 mean pain score reduction in patients that received prochlorperazine bundle therapy as compared to those that received droperidol bundle therapy, it is difficult to ascertain if prochlorperazine is more efficacious, as the droperidol bundle group experienced delays between medication administration and pain scores. While our findings were unable to support a statistically significant difference in pain score reduction between the three treatment groups, they do affirm that both droperidol and prochlorperazine are effective at decreasing migraine-related pain without significantly different adverse outcomes.

The droperidol monotherapy group was associated with higher rates of rescue therapy, inpatient admission, and ED length of stay, as well as a lower mean pain score reduction as compared to the other treatment groups. While not statistically significant, this may demonstrate the value of NSAIDs as a first-line agent in the management of migraines [3].

One limitation of this study was that it was underpowered, such that statistically significant differences were not detected if they did exist. A high volume of patients was excluded due to a lack of pain score charting, resulting in small treatment arms. Additionally, while the three treatment arms were mostly similar at baseline, there was a statistically significant difference in age across the three arms. Given that this study was conducted retrospectively, prior to admission medications were unable to be fully screened for and chart review was reliant on accurate documentation.

While concomitant administration of most antimigraine and analgesic medications met criteria for exclusion, some medications, such as acetaminophen, diphenhydramine, and magnesium, were concomitantly administered but not excluded, as these are adjunctive therapies and not necessarily considered standard of care for migraine treatment. Conversely, while diphenhydramine is commonly included in migraine treatment bundles, we opted not to include this agent as one of the study medications in our bundled treatment arms, as diphenhydramine is sometimes omitted from migraine therapy. Adjunctive medications included acetaminophen (54.4%), diphenhydramine (32.9%), magnesium (24.1%), lidocaine patches (3.8%), lorazepam (2.5%), aspirin (1.3%), ketamine (1.3%), and dexamethasone (1.3%).

## Conclusions

We found no statistically significant differences in efficacy at reducing migraine-related pain, emergency department length of stay, rates of inpatient admission, rates of rescue therapy, or adverse events between droperidol monotherapy and droperidol- and prochlorperazine-based therapies. While a larger pain score reduction was observed in the prochlorperazine bundle treatment group, this finding was not statistically significant and obscured by an increased delay between medication administration and pain score documentation in the droperidol bundle treatment group. Prospective research should be conducted to assess the efficacy of droperidol in the treatment of migraines with larger sample sizes and predefined timing between pain score charting and medication administration.
